# Improving oxygenation in severe ARDS treated with VV-ECMO: comparative efficacy of moderate hypothermia and landiolol in a swine ARDS model

**DOI:** 10.1186/s40635-024-00655-2

**Published:** 2024-08-27

**Authors:** Maud Vincendeau, Thomas Klein, Frederique Groubatch, N’Guyen Tran, Antoine Kimmoun, Bruno Levy

**Affiliations:** 1 Service de Médecine Intensive et Réanimation Brabois, Université de Lorraine, CHRU de Nancy, Nancy, France; 2 Université de Lorraine, INSERM U1116, Nancy, France; 3 Université de Lorraine, CHRU de Nancy, Service d’Anesthésie Réanimation de Chirurgie Cardiaque et Transplantation, Nancy, France; 4 Université de Lorraine, École de Chirurgie, Nancy, France

**Keywords:** Acute Respiratory distress syndrome, Extracorporeal membrane oxygenation, Hypothermia, Adrenergic beta-antagonists

## Abstract

**Background:**

Acute respiratory distress syndrome (ARDS) remains a significant challenge in critical care, with high mortality rates despite advancements in treatment. Venovenous extracorporeal membrane oxygenation (VV-ECMO) is employed as salvage therapy for refractory cases. However, some patients may continue to experience persistent severe hypoxemia despite being treated with VV-ECMO. To achieve this, moderate hypothermia and short-acting selective β1-blockers have been proposed.

**Methods:**

Using a swine model of severe ARDS treated with VV-ECMO, this study investigated the efficacy of moderate hypothermia or β-blockade in improving arterial oxygen saturation (SaO_2_) three hours after VV-ECMO initiation. Primary endpoints included the ratio of VV-ECMO flow to cardiac output and arterial oxygen saturation before VV-ECMO start (H0) and three hours after ECMO start (H3). Secondary safety criteria encompassed hemodynamics and oxygenation parameters.

**Results:**

Twenty-two male pigs were randomized into three groups: control (*n* = 6), hypothermia (*n* = 9) and β-blockade (*n* = 7). At H0, all groups demonstrated similar hemodynamic and respiratory parameters. Both moderate hypothermia and β-blockade groups exhibited a significant increase in the ratio of VV-ECMO flow to cardiac output at H3, resulting in improved SaO_2_. At H3, despite a decrease in oxygen delivery and consumption in the intervention groups compared to the control group, oxygen extraction ratios across groups remained unchanged and lactate levels were normal.

**Conclusions:**

In a swine model of severe ARDS treated with VV-ECMO, both moderate hypothermia and β-blockade led to an increase in the ratio of VV-ECMO flow to cardiac output resulting in improved arterial oxygen saturation without any impact on tissue perfusion.

## Introduction

Acute respiratory distress syndrome (ARDS) is an acute inflammatory lung injury, characterized by increased pulmonary vascular permeability and loss of aerated lung tissue [1]. Despite medical advancements, severe ARDS still exhibits a high mortality rate, reaching up to 50% [2]. Venovenous extracorporeal membrane oxygenation (VV-ECMO) serves as a last-resort treatment when conventional methods prove insufficient [3]. However, even with VV-ECMO, some patients may still experience very low levels of arterial oxygen saturation (SaO_2_), which are potentially life-threatening [4]. One major pathophysiological explanation is the inadequate ratio of VV-ECMO flow to cardiac output (Q_ECMO_/CO) where the CO is too high relative to VV-ECMO flow. Schmidt et al. demonstrated that a Q_ECMO_/CO ratio greater than 60% should be targeted to achieve a SaO_2_ greater than 90% [5]. If a patient remains severely hypoxemic despite a high Q_ECMO_ with minimal recirculation and an adequate hemoglobin level, strategies to reduce the CO might be considered. Proposed bedside interventions might include moderate hypothermia or the use of β-blockers to improve Q_ECMO_/CO [6, 7]. Hypothermia, widely studied for the management of post-cardiac arrest patients is known to reduce heart rate and CO but also increases the onset of infections and arrhythmias [8]. An alternative option might be the use of β-blockers, such as landiolol, an ultra-short-acting β1 selective adrenergic antagonist, which could also decrease heart rate and CO but has negative inotropic effects and thus could potentially reduce oxygen delivery (DO_2_) [9].

Using a swine model of ARDS, this study aims to investigate the efficacy of moderate hypothermia and β-blockade within the first three hours post-VV-ECMO initiation on Q_ECMO_/CO and arterial oxygen saturation (SaO_2_).

## Methods

### Animal preparation

Twenty-four male pigs (Landrace) weighing between 45 and 60 kg and aged nine months were used in this study. They were housed in groups under controlled conditions within a certified animal facility. A quarantine period of at least eight days was required before the animals entered the protocol to stabilize their physiological and behavioral states and to improve the experimental conditions.

### Anesthesia procedure

After a one-day fast, the animals were placed under general anesthesia and ventilation. Anesthesia was induced via the lateral auricular vein with an intravenous bolus of propofol (1 mg.kg^−1^, Propofol-lipuro 1%, B. Braun, Melsungen AG, Germany). The animals were then intubated (TeleflexIsis 7.5 I.D. mm, Teleflex Medical, Athlone, Ireland) and mechanically ventilated (Evita 1 Dura, Dräger, Luebeck, Germany) in assisted-controlled mode (30% oxygen, tidal volume 10 ml.kg^−1^, and respiratory rate 12 min^−1^). Anesthesia was maintained with a continuous infusion of sufentanil (0.2 μg.kg^−1^.min^−1^, Sufentanil, Mylan, Canonsburg, Pennsylvania, USA), propofol (7 mg.kg^−1^.h^−1^, propofol-lipuro 2%, B. Braun Melsungen AG, Germany), and cisatracurium (0.9 mg.kg^−1^.h^−1^, Nimbex, GlaxoSmithKline, Brentford, Middlesex, UK).

### Catheter placement

The animals were monitored by continuous electrocardiogram recording. An arterial catheter (7 Fr, Seldicath, Plastimed Prodimed ®, France) was inserted into the left carotid artery for continuous systemic arterial pressure measurement. A triple lumen catheter (8 Fr, Arrow ®, Reading, USA) was inserted into the left external jugular vein. After a low median laparotomy, a vesical catheter was placed. Core body temperature was measured via a rectal probe and maintained at 38.5 °C, which corresponds to the physiological body temperature in pigs. The temperature was regulated using a thermal management unit connected to the ECMO circuit.

### ECMO implantation

The cannulas were introduced using the Seldinger technique following repeated dilatations of the targeted veins. The right external jugular vein was dissected, and a return cannula (17 Fr, Biomedicus cannulae, Medtronic, USA) was inserted. The venous drainage cannula (21 Fr, Biomedicus cannulae, Medtronic ®, USA) was introduced percutaneously under ultrasound control through the right common femoral vein. Their positions were assessed by transesophageal echography and by fluoroscopy. The procedure was followed by the administration of a 100 UI/kg dose of unfractioned heparin (Heparin Sodique Choay, Sanofi-Aventis, Paris, France). Distance between the cannulas were set at 10 to 13 cm to prevent recirculation phenomenon. The device consisted of a console (Rotaflow Console, Maquet, Germany), a centrifugal pump (Rotaflow Centrifugal Pump System, Maquet, Germany), a tubing, along with the membrane oxygenator (PLS-I Oxygenator, Maquet, Germany), which was connected to a mechanical gas blender system (Sechrist Model 20090, Sechrist, USA). The fully assembled ECMO circuit was primed with saline solution (NaCl 0.9%, B. Braun Medical, France).

### Acute respiratory distress syndrome model

The ARDS model employed a double-hit approach as previously published [10]. Briefly, this method combined surfactant depletion with saline washout until gas exchange and pulmonary compliance were significantly impacted. Surfactant depletion was achieved through repeated alveolar washouts using warm 0.9% saline (30 ml.kg^−1^). Barotraumatic ventilation was performed using pressure-controlled ventilation with a positive end expiratory pressure (PEEP) of 0 cmH_2_O, an inspiratory pressure of 40 cmH_2_O, a respiratory rate of 10 min^−1^, and an inspiratory to expiratory ratio of 1:1. This “traumatic” ventilation was maintained for at least one hour and up to two hours. VV-ECMO started when partial pressure of oxygen in arterial blood (PaO_2_) dropped below 80 mmHg on fraction of inspired oxygen (FiO_2_) at 100%.

#### ECMO settings

- ECMO output based on a theoretical cardiac output of 70 ml.kg^−1^.min^−1^.
- Membrane oxygen fraction was set at 100%.
- Initial sweep gas flow was 3 L.min^−1^, and then adjusted to target a partial pressure of carbon dioxide in arterial blood (PaCO_2_) between 35 and 45 mmHg.
- Ventilator settings were a tidal volume of 2 ml.kg-1, a respiratory rate of 10 /min, a PEEP between 10 and 15 cmH_2_O, maintaining a plateau pressure below 25 mmHg, and an FiO_2_ of 60%.

#### Groups

- **Control group:** Temperature was maintained at 38.5 °C.
- **Moderate hypothermia group:** Temperature was lowered using a heater-cooler unit (HCU20 Getinge-Maquet, Germany) by one degree every 15 min until the **QECMO/CO** ratio reach 60% (minimum temperature was 34 °C).
- **β-blocker group:** Landiolol (Rapibloc, 300 mg, Amomed, France) infusion started at 1 µg.kg^−1^.min^−1^ and was increased by one µg.kg^−1^.min^−1^ every five minutes until the **QECMO/CO** reaches 60% (maximum dose of 20 µg.kg^−1^.min^−1^).

### Timing of the measurements

Data collection was performed after surgery but prior to ARDS induction, after ARDS induction and before VV-ECMO initiation (H0), and 3 h post-ARDS initiation with animals treated by VV-ECMO (H3).

### Hemodynamic measurements

Heart rate, systolic, mean and diastolic arterial pressures were continuously recorded. Arterial lactate and hemoglobin levels were measured at the times of blood gas. Maximal norepinephrine dosage was recorded. From transesophageal echography, CO was obtained from the average of three measures using the diameter of the aortic ring, the subaortic velocity time integral (VTI), and the heart rate. The formula used was:

$$CO ({l.min}^{-1})=sub\, aortic\, VTI (cm) \times HR {(min}^{-1}) \times \text{3,14}\times {\left(\frac{Aortic\, ring\, diameter \left(cm\right)}{2}\right)}^{2}.$$

Oxygen delivery (DO_2_) was calculated according to the following formula:

Arterial oxygen content (CaO_2_):

$${CaO}_{2}({ml.dl}^{-1})=\frac{1.34\times Hemoglobin\, level \left({g.dl}^{-1}\right)\times {SaO}_{2} \left(\%\right)}{100}+{PaO}_{2} (mmHg)\times 0.003).$$

Venous oxygen content (CvO_2_):

$${CvO}_{2}({ml.dl}^{-1})=\frac{1.34\times Hemoglobin\, level ({g.dl}^{-1})\times {SvO}_{2} (\%)}{100}+{PvO}_{2} (mmHg)\times 0.003$$

$${DO}_{2} \left({ml.min}^{-1}\right)=10 \times CO \left({L.min}^{-1}\right)\times {CaO}_{2}\left({ml.dl}^{-1}\right).$$

Oxygen consumption (VO_2_):

$${VO}_{2}\left({ml.min}^{-1}\right)=10 \times CO \left({L.min}^{-1}\right)\times {(CaO}_{2}\left({ml.dl}^{-1}\right)-{CvO}_{2}({ml.dl}^{-1}))$$

Oxygen extraction rate (OER):

$$OER \left(\%\right)=\frac{{CaO}_{2}({ml.dl}^{-1})-{CvO}_{2}({ml.dl}^{-1})}{{CaO}_{2}({ml.dl}^{-1})}.$$

### Hemodynamic management

In case of vascular collapse at VV-ECMO initiation or unexpected drops in VV-ECMO flow or cannula shaking, 500 ml of 0.9% NaCl was administered and could be repeated up to four times. If necessary, norepinephrine was allowed to maintain the mean arterial pressure between 65 and 70 mmHg.

### Respiratory data collection

Respiratory parameters including tidal volume, positive end expiratory pressure, respiratory rate, plateau pressure, driving pressure and FiO_2_ were collected. Blood gas was collected including pH, PaO_2_, SaO_2_, and PaCO_2_.

### Procedure

After animal surgery (catheters and cannulas placements), hemodynamic data were collected, and the ARDS procedure was initiated. When ARDS criteria were met (PaO_2_ dropped below 80 mmHg on FiO_2_ 100%), hemodynamic, biological and ventilatory parameters were collected (H0). Then, the animals were randomly assigned to one of the three predefined group, and VV-ECMO was initiated. Interventions were maintained for three hours, and a final data collection was performed.

### End of experiment

Animals were euthanized after H3 measurements with pentobarbital (approximately 10 mL, Exagon, Axience, France).

### Statistical method

Continuous data were described using the median, first quantile, and third quantile. For characteristics prior to and after ARDS induction, Kruskal–Wallis tests were conducted. Comparisons over time were performed using a linear mixed model that incorporated a random effect for each pig. The normality of the residuals was assessed both graphically and via the Shapiro–Wilk test. When assumptions were not met, paired Wilcoxon and Kruskal–Wallis tests were applied as appropriate and a post hoc Dunn test could be applied when necessary. A two-sided alpha risk was set at 0.05. All analyses were conducted using R version 4.1.1 (2021-08-10).

## Results

A total of 24 animals were initially included. However, two pigs died from hemorrhagic shock during the cannulation process. Thus, 22 animals were ultimately included in the study, divided into three groups after randomization: control (*n* = 6), hypothermia (*n* = 9) and β-blocker (*n* = 7). Except for the weight, the three groups were similar for all hemodynamic, respiratory and biological parameters before ARDS induction (Table 1).

**Table 1:** Characteristics after surgery and before ARDS induction in the three groups

variables	*N*	Control	*N*	Hypothermia	*N*	β-blocker	*p*-value
General parameters							
Weight (kg)	6	60 (56–60)	9	53 (52–57)	7	54 (52–55)	0.013
Central temperature(℃)	6	38.0 (37.6–38.3)	9	37.7 (37.5–38.0)	7	38.0 (37.1–39.1)	0.64
Hemoglobin level (g.dl^−1^)	6	11.0 (10.3–11.7)	9	9.5 (8.8–10.7)	7	10.7 (10.0–12.1)	0.093
Hemodynamic parameters							
Mean arterial pressure (mmHg)	6	74 (71–78)	9	73 (69–83)	7	72 (70–83)	0.97
Heart rate (min^−1^)	6	106 (100–115)	9	122 (120–148)	7	114 (92–125)	0.043
Cardiac output (l.min^−1^)	6	6.2 (5.4–7.0)	9	6.5 (6.1–7.5)	7	5.6 (5.3–6.4)	0.13
Lactate (mmol.l^−1^)	6	0.6 (0.4–1.0)	9	1.2 (0.5–1.6)	7	1.1 (0.7–1.2)	0.19
Norepinephrine dose (γg.kg^−1^.min^−1^)	6	0 (0–0)	9	1 (0–2)	7	0 (0–1)	0.25
*Fluid expansion (ml)	6	450 (400–500)	9	1000 (600–2000)	7	750 (0–1000)	0.090

Data are expressed as median, 25 e and 75e quantile. *Sum of fluids administered during the surgery

### Model characterization after ARDS induction and before VV-ECMO start (H0) (Table 2)

**Table 2:** Characteristics after ARDS induction and before VV-ECMO start in the three groups

Variables	*N*	control	*N*	hypothermia	*N*	β-blocker	*p*-value
General parameters							
Central temperature(℃)	6	38.7 (38.6–38.8)	9	38.3 (38.0–38.5)	7	38.4 (38.2–38.5)	0.005
Hemoglobin level (g.dl^−1^)	6	10.4 (9.7–10.7)	9	9.0 (8.6–10.0)	7	9.5 (9.0–10.2)	0.15
Hemodynamic parameters							
Mean arterial pressure (mmHg)	6	84 (78–85)	9	69 (66–72)	7	70 (65–70)	0.012
Heart rate (bpm)	6	138 (124–142)	9	153 (136–174)	7	138 (133–140)	0.22
Cardiac output (l.min^−1^)	6	7.9 (7.5–8.7)	9	8.2 (7.5–9.4)	7	7.6 (7.3–8.0)	0.28
Lactate (mmol.l^−1^)	6	1.4 (0.9–1.5)	9	1.0 (0.8–1.7)	7	1.8 (1.0–2.1)	0.65
Norepinephrine dose (γg.kg^−1^.min^−1^)	6	0.5 (0.4–0.6)	9	1.1 (0.8–4.5)	7	0.4 (0.3–1.0)	0.019
*Fluid expansion (ml)	6	500 (500–1000)	9	900 (750–1000)	7	1000 (1000–1500)	0.041
Respiratory parameters							
Sao_2_ (%)	6	74 (67–80)	9	79 (78–85)	7	77 (72–81)	0.14
Pao_2_/fio_2_	6	48 (44–55)	9	57 (55–60)	7	54 (45–60)	0.14
Fio_2_ (%)	6	100 (100–100)	9	100 (100–100)	7	100 (100–100)	-
Static compliance (ml.cmh_2_O^−1^)	6	24 (23–25)	9	21 (17–24)	7	20 (19–20)	0.048

Data are expressed as median, 25 e and 75e quantile. *Sum of fluids administered during the ARDS procedure

At H0, the PaO_2_ to FiO_2_ ratios were similar between the three groups (*p* = 0.14) as the decrease in SaO_2_ with control: 74 (67–80) %, hypothermia: 79 (78–85) % and β-blocker: 77 (72–81) %, (*p* = 0.14). Static respiratory system compliance was also roughly similar across groups with control: 24 (23–25) ml.cmH_2_O^−1^, hypothermia: 20 (17–24) ml.cmH_2_O^−1^ and β-blocker: 20 (19–20) ml.cmH_2_O^−1^, (*p* = 0.048). Cardiac output was also similar between groups with control: 7.9 (7.5–8.7) l.min^−1^, hypothermia: 8.2 (7.5–9.4) l.min^−1^and β-blocker: 7.6 (7.3–8) l.min^−1^ (*p* = 0.28).

### Hemodynamic and oxygenation parameters comparison between groups before and three hours after ARDS initiation

At H3, in the β-blocker group the median and β-blocker (landiolol) dose was 14 (11–17) $\gamma$ g.kg^−1.^min^−1^ an in the hypothermia group, at H3 the median temperature was 34.5 (34.3–34.9) °C.

### VV-ECMO flow-to-cardiac output ratio

**Fig. 1:**
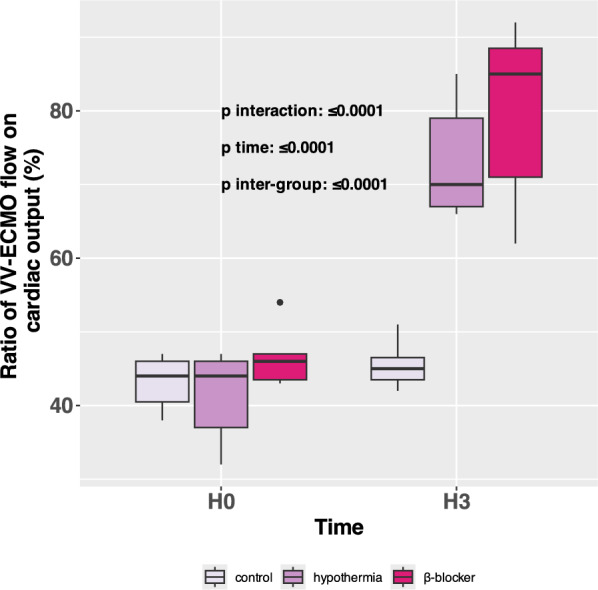
Ratio of VV-ECMO flow to cardiac output in percent in the three groups after ARDS induction and before VV-ECMO initiation (H0) and three hours post-ARDS initiation treated by VV-ECMO (H3)

At H0, QECMO/CO were similar across the three groups. At H3, there was an increase in ratios in hypothermia and β-blocker groups compared to H0, while control remained at the same value (control: from 44 (41–46) % at H0 to 45 (44–47) % at H3, hypothermia: from 44 (37–46) % to 70 ( 67–79) %, at H3 β-blocker: from 46 (44–47) % to 85 ( 71- 89) %, p interaction $\le$ 0.0001) **(**Fig. 1**).**

### Arterial oxygen saturation

**Fig. 2:**
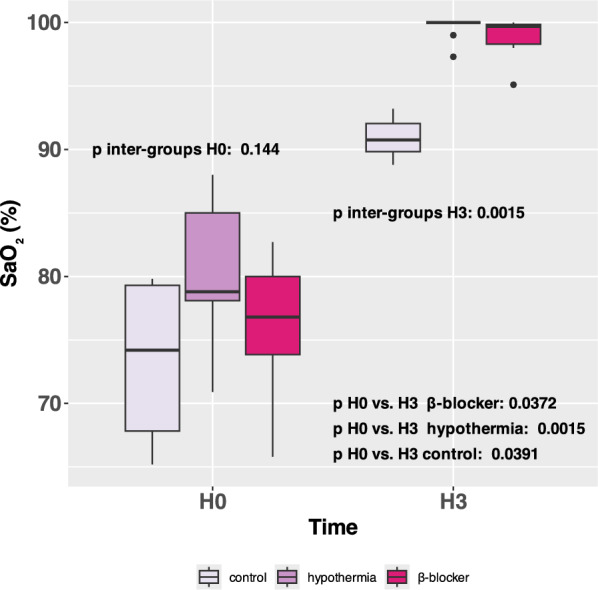
Arterial oxygen saturation (SaO_2_) in percent in the three groups after ARDS induction and before VV-ECMO initiation (H0) and three hours post-ARDS initiation treated by VV-ECMO (H3)

At H0, the SaO_2_ were similar across the three groups event if the SaO_2_ tended to be lower in the control group (p inter-group H0 = 0.114). At H3, there was an increase in SaO_2_ in the three groups compared to H0 but this increase was higher in the two intervention groups (control: from 74 ( 68–79) % at H0 to 91 ( 90–92) % at H3, hypothermia: from 79 (78–85) % to 100 (100–100) %, at H3 β-blocker: from 77 (74–80) % to 100 (98–100) %, p values H0 vs H3 < 0.005 for each group) **(**Fig. 2**).**

### Oxygen delivery, consumption and extraction ratio

**Fig. 3:**
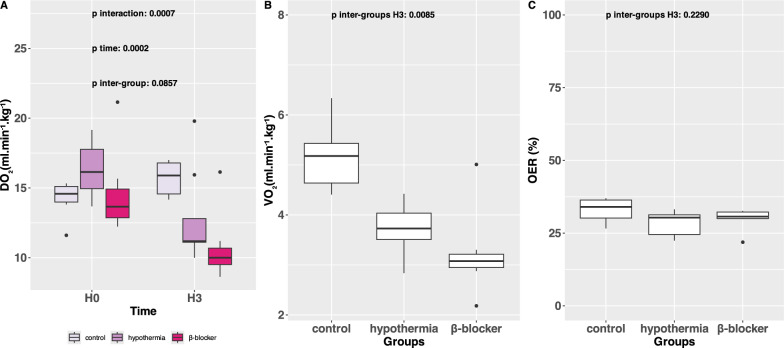
Oxygen delivery (DO_2_) in the three groups after ARDS induction and before VV-ECMO initiation (H0) and three hours post-ARDS initiation treated by VV-ECMO (H3), oxygen consumption (VO_2_) and oxygen extraction ratio in the three groups, three hours post-ARDS initiation treated by VV-ECMO (H3). **A** DO_2_ (ml.min^−1^.kg^−1^), Panel B: VO_2_ (ml.min^−1^.kg^−1^), **C** OER (%), *n* = 5/9 in the hypothermia group for **B** and **C**

At H0, DO_2_ was similar between the control and hypothermia groups, with a trend towards a decrease in the β-blocker group compared to the other two (p = 0.21). At H3, DO_2_ decreased in the intervention groups compared to the control group and to H0 (at H3, control: 15.9 (14.6–16.8) ml.min^−1^.kg^−1^, hypothermia: 11.2 (11.1- 12.8) ml.min^−1^.kg^−1^, β-blocker: 10 (9.51- 10.7), ml.min^−1^.kg^−1^, p interaction = 0.0002, Fig. 3, panel A). At H3, VO_2_ was decreased in intervention groups compared to the control group (control 5.2 (4.6- 5.4) ml.min^−1^.kg^−1^, hypothermia: 3.7 (3.5- 4.0) ml.min^−1^.kg^−1^, β-blocker: 3.1 (2.9- 3.2), ml.min^−1^.kg^−1^ *p* = 0.0031) with no difference between the hypothermia and β-blocker groups (*p* = 0.403, Fig. 3, panel B). Oxygen extraction ratios were similar at H3 between groups (*p* = 0.403, Fig. 3, panel C).

#### Safety criteria

**Fig. 4:**
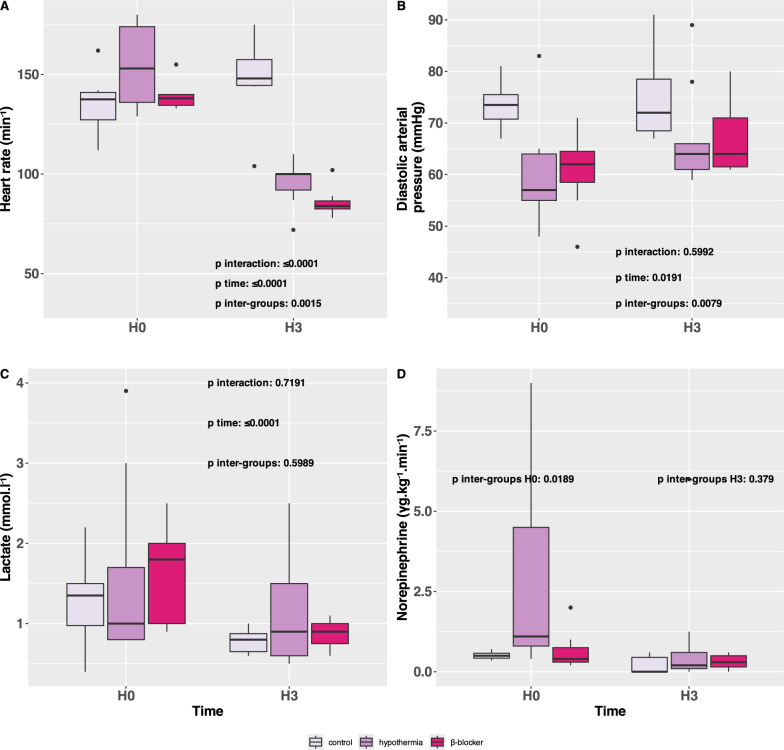
Safety parameters in the three groups after ARDS induction and before VV-ECMO initiation (H0) and three hours post-ARDS initiation treated by VV-ECMO (H3). **A** Heart rate (min^−1^), **B** diastolic arterial pressure (mmHg), **C** lactate (mmol.l^−1^), **D** norepinephrine dose ($\gamma$ g.kg^−1^.min.^−1^)

No arrhythmia was observed during the 3-h protocol. The heart rate was reduced in the intervention groups (Fig. 4, panel A). There were no significant differences observed between groups in terms of diastolic arterial pressure (Fig. 4, panel B), norepinephrine dosage (Fig. 4, panel C) and lactate levels (Fig. 4, panel D) during the protocol.

## Discussion

Using a well-established swine model of ARDS treated with VV-ECMO, both β-blocker and moderate hypothermia improve arterial oxygen saturation compared to a control group. This improvement is attributed to an increase in the ratio of QECMO/CO. Notably, no adverse hemodynamic, oxygenation or arrhythmic events were observed during the protocol, highlighting the interventions' safety.

### ARDS model

Our model utilizes surfactant depletion and traumatic ventilation to induce ARDS, methods validated in previous literature which also includes endotoxin administration and smoke inhalation as alternative models [10, 11]. Although our approach does not perfectly mirror the clinical pathophysiological mechanisms of ARDS, it effectively simulates surfactant depletion combined with pulmonary hypertension, offering valuable insights into the efficacy of VV-ECMO in severe cases. The model's relevance and accuracy have been confirmed by prior studies [12].

### Efficacy of moderate hypothermia and β-blocker in improving arterial oxygen saturation

The implementation of moderate hypothermia or the administration of β-blockers in this study led to a reduction in CO, primarily due to a decrease in heart rate. This resulted in an improvement in the QECMO/CO ratio, surpassing the 60% threshold considered optimal for effective gas exchange. By improving QECMO/CO, the fully oxygenated blood provided to the venous circulation by QECMO adequately met the native CO improving drastically the SaO_2,_ as it was already demonstrated by others. Moreover, these findings could be associated with additional potential benefits, such as reducing VV-ECMO flow, which in turn may lower the risk of hemolysis. The latter has been found to be more common in non-survivors of VV-ECMO and could elevate the risk of renal failure [13].

### Oxygen delivery as matter of concern with hypothermia and β-blocker interventions

We observed a reduction in DO_2_ in the intervention groups while SaO_2_ drastically increased. This result is conflicting: on one hand, SaO_2_ improved, but on the other hand, DO_2_ was reduced. This reduction in DO_2_ could imply a potential worsening of tissue hypoxia. However, in this study, no increase in lactate levels or hemodynamic instability was found three hours post-ARDS induction in the intervention groups. Some may argue that the exposure time was too short to observe a worsening of a potential tissue hypoxia. Based on these findings, some authors strongly advise against the use of β-blockers in ARDS patients treated with VV-ECMO [7, 14]. However, three major counterarguments fuel this ongoing debate. First, in this study, we compare two interventions in VV-ECMO treated ARDS: hypothermia and β-blockers, against a control group. Moderate hypothermia has a hemodynamic profile, in our study, similar to that of β-blockers. Heart rate and CO are reduced equally in both intervention groups, resulting in a roughly similar reduction in DO_2_ and VO_2_. A notable observation is that moderate hypothermia, whether in cardiac arrest or cardiogenic shock, is not associated with any deleterious effect on tissue perfusion [15]. Second, the metabolic effects of moderate hypothermia also induce a global VO_2_ reduction, and some experimental studies point out that tissue partial oxygen pressure is not significantly altered [16]. β-blockers also reduce VO_2_, but mainly by reducing myocyte oxygen consumption. The latter represents around 10% of total body oxygen consumption (6–8 ml.100g^−1^.min^−1^) in physiological conditions and is increased in case of sympathetic activation in shock states [17]. However, at bedside, VO_2_ is not routinely measured. Third, in the present study, although VO_2_ values were reduced in both intervention groups, OER remained constant across the three groups, which is reassuring. Nevertheless, caution is warranted when using β-blockers in ARDS patients treated with VV-ECMO to adapt the QECMO/CO ratio, specifically in cases of hemodynamic instability, as β-blockers could potentially compromise the minimal DO_2_ essential for patient recovery. Close monitoring, including clinical examinations focused on the onset of mottling or prolongation of capillary refill time, measurements of lactate levels, hemoglobin levels, and iterative measures of CO via transthoracic echocardiography, should be performed to detect an imbalance in VO_2_/DO_2_ early. Indeed, patients treated with VV-ECMO are at high risk of serious bleeding and hemolysis leading to blood transfusion [18]. In our study, all animals were treated with neuromuscular blockers to ensure adaptation to protective ventilation and to prevent the onset of shivering in the hypothermia group. This use may have reduced VO_2_ in addition to the tested interventions and, to a certain extent, DO_2_ [19–21]. The literature is conflicting regarding the usefulness of neuromuscular blockers in ARDS patients [22–25]. On one hand, neuromuscular blockers enhance thoraco-pulmonary compliance, alveolar recruitment, and may prevent biotrauma and reduce oxygen consumption. On the other hand, they are associated with ICU-acquired weakness and necessitate deep sedation, with no proven benefit on ventilator-free days or 90-day outcome. The use of hypothermia in ARDS patients supported by VV-ECMO almost always requires neuromuscular blockers to prevent shivering, which is a significant clinical consideration compared to the β-blocker approach that does not.

### Short-term outcomes and further research

There are already several case series of patients with severe hypoxemia despite being treated with a VV-ECMO, in whom moderate hypothermia or β-blockers have been successfully tested [9, 26–33]. Our short-term experimental results are in line with these results, showing minimal side effects, but clinical studies with prolonged exposures to interventions are warranted to confirm the efficiency and the safety profile. The ongoing HYPOLUNGECMO clinical trial aimed to investigate the effectiveness of induced moderate hypothermia in patients with severe ARDS treated with VV-ECMO (NCT05306392, last registered on April 1, 2022).

### Study limitations

First, our study was conducted on healthy young pigs, which do not have the pre-existing cardiac or pulmonary comorbidities commonly found in ARDS patients. Second, since the duration of the protocol was only three hours, the long-term effects were not explored. ARDS management usually lasts for several days or even weeks [34]. Third, our model did not consider other crucial therapeutic approaches such as prone positioning. While prone positioning is essential for the management of ARDS patients with severe hypoxemia [1], recent data demonstrated that its systematic and early use in ARDS patient treated with VV-ECMO is not associated with a survival benefit. However, this study included almost exclusively a population of COVID-19 ARDS patients [35]. Finally, the use of transesophageal echocardiography for CO measurement, as no other technique is validated in animals treated with VV-ECMO, introduces a potential operator-dependent bias. We attempted to mitigate this by having all measurements performed in triplicate by a single, well-trained operator.

## Conclusion

In an experimental model of ARDS treated with VV-ECMO, hypothermia and β-blockers are effective interventions for improving the ECMO flow to cardiac output ratio and arterial oxygen saturation without adverse effects on macrocirculation or tissue perfusion. Both interventions show promise in optimizing oxygenation in persistent severe hypoxemia in ARDS patients treated with VV-ECMO. Further clinical studies are needed to confirm these benefits in real-world cases and to explore optimal dosing, timing, and patient selection.

## Disclosure

Chat GPT (Open AI) was used to check spelling and grammar.

## Data Availability

On reasonable request, by e-mail to Pr Levy, blevy5463@gmail.com.

## Abbreviations

ARDS: Acute respiratory distress syndrome
CO: Cardiac output
DO_2_: Oxygen transport
FiO_2_: Fraction of inspired oxygen
QECMO/CO: VV-ECMO flow-to-cardiac output ratio
PaCO_2_: Partial pressure of carbon dioxide in arterial blood
PaO_2_: Partial pressure of oxygen in arterial blood
SaO_2_: Arterial oxygen saturation
VO_2_: Oxygen consumption
VV-ECMO: Venovenous extracorporeal membrane oxygenation
